# Correlation of quantitative MRI and neuropathology in epilepsy surgical resection specimens—T2 correlates with neuronal tissue in gray matter

**DOI:** 10.1016/j.neuroimage.2007.04.051

**Published:** 2007-08-01

**Authors:** S.H. Eriksson, S.L. Free, M. Thom, L. Martinian, M.R. Symms, T.M. Salmenpera, A.W. McEvoy, W. Harkness, J.S. Duncan, S.M. Sisodiya

**Affiliations:** Department of Clinical and Experimental Epilepsy, Institute of Neurology, University College London, Queen Square, Box 29, London WC1N 3BG, UK

**Keywords:** Epilepsy, MRI, Histology, Fast Flair T2, MTR

## Abstract

Newer MRI methods can detect cerebral abnormalities not identified on routine imaging in patients with focal epilepsy. Correlation of MRI with histopathology is necessary to understand the basis of MRI abnormalities and subsequently predict histopathology from in vivo MRI. The aim of this study was to determine if particular quantitative MR parameters were associated with particular histological features.

Nine patients with temporal lobe epilepsy were imaged at 1.5 T using standard presurgical volumetric and quantifiable sequences: magnetization transfer and FFT2. The resected temporal lobe was registered with the volumetric MRI data according to our previously described method to permit correlation of the modalities. Stereologically measured neuronal densities and field fraction of GFAP, MAP2, synaptophysin and NeuN immunohistochemistry were obtained. Analyses were performed in the middle temporal gyrus and compared with quantitative MRI data from the equivalent regions.

There was a significant Spearman Rho negative correlation between NeuN field fraction and the T2 value in gray matter (correlation coefficient − 0.72, *p* = 0.028). There were no significant correlations between any neuropathological and MR measures in white matter.

These preliminary findings suggest that T2 in gray matter is sensitive to the proportion of neuronal tissue. Novel quantitative MRI measures acquired with higher field strength magnets, and so with superior signal to noise ratios, may generate data that correlate with histopathological measures. This will enable better identification and delineation of the structural causes of refractory focal epilepsy, and will be of particular benefit in patients in whom current optimal MRI does not identify a relevant abnormality.

## Introduction

The structural basis of focal epilepsy has a major effect on the natural history of the condition in an individual patient (Semah et al., 1998). In 30% of patients, epilepsy is refractory to current drug treatments. In patients with drug-resistant focal epilepsy, surgical resection may be curative, and the nature of the underlying lesion influences post-operative outcome (McIntosh et al., 2001; Luders and Schuele, 2006).

MRI has greatly facilitated assessment for epilepsy surgery by revealing underlying structural cerebral abnormalities. When optimal MRI and post-operative neuropathological examination of the resection specimen do not identify an underlying structural abnormality, seizure outcome from surgery is less likely to be good (Blume et al., 2004; Jeha et al., 2006), reflecting the absence of a discrete, removed abnormality or one that is currently undetectable by imaging or pathology. This is to some extent a self-fulfilling strategy, such that patients in whom no structural abnormality is identified may not be considered for surgery (Spencer et al., 2005). Even optimal MRI, however, may not reveal known underlying pathological substrates such as focal cortical dysplasia and mild malformations of cortical development (Palmini et al., 2004), which are evident on histopathological examination of the resected tissue (Lee et al., 2005; Widdess-Walsh et al., 2006).

Thus, there is a pressing need for further developments in MRI, such as higher field strengths to improve signal-to-noise ratio, and newer sequences that may better pick out culpable neuropathological lesions in the neocortex.

The correlations between hippocampal volumes and T2 measures with glial and neuronal densities were established more than a decade ago (Cascino et al., 1991; Van Paesschen et al., 1995). For the more recently developed MR contrasts, the correlation between MR parameters and the underlying neocortical structure is not well understood and it is important for this to be clarified if these contrasts are to contribute to presurgical evaluation.

Quantitative histopathology has developed in parallel with MRI and led to more accurate and sophisticated interpretation of microscopic tissue structure (Thom et al., 2005). We have previously shown that temporal lobe resection specimens and their resultant histological slides can be aligned with pre-operative volumetric MRI data, allowing comparison of advanced MRI data sets in regions of interest to the equivalent areas on histopathological slides (Eriksson et al., 2005). Thus, quantitative MRI data and quantitative histopathological data can be directly compared. In this study we compared the data obtained using two quantitative MRI contrasts, T2 relaxation time maps (T2); and magnetization transfer ratio (MTR), with quantitative neuropathological data from the resected material. The aim was to explore the clinical utility of T2 and MTR and determine if particular quantitative MR parameters were associated with particular histological features.

## Methods

### Subjects

We studied nine consecutive patients for temporal lobe epilepsy surgery at the National Hospital for Neurology and Neurosurgery who had had pre-operative advanced MRI and in whom resection specimens were processed according to our protocol (Eriksson et al., 2005). The study was approved by the Joint Research Ethics Committee of the National Hospital for Neurology and Neurosurgery and Institute of Neurology, UCL. All patient and control subjects gave written informed consent to participate in the study.

The age range of subjects was 31–46 years (median 36 years) and 4 were male. Seven had a right anterior temporal lobe resection, and two had surgery on the left. The surgical procedure was selected after our standard presurgical work-up, including EEG-video telemetry, conventional MRI (T1- and T2-weighted, proton density and FLAIR), psychological and psychiatric assessments and multi-disciplinary case conference.

Control groups for the advanced MRI consisted of healthy volunteers with no history of neurological disease and normal conventional MRI and were the same subjects as reported previously (Salmenpera et al., 2007). Twenty-nine volunteers served as a control population for the analysis of T2 maps and 26 volunteers for MTR.

### MR acquisition

All scans were acquired on the same 1.5 T GE Signa MR scanner (GE Medical Systems, Milwaukee, WI). The T1-weighted volume sequence from the conventional scan protocol for all subjects was required for the MR:pathology correlation. The sequence parameters were: TE/TR/TI/NEX, 4.2/15/450/1; flip angle 20°; acquisition matrix 256 × 192; field of view 24 × 18 cm; 3/4 phase FOV, 124 contiguous 1.5 mm slices, giving a voxel size of 0.94 × 1.25 × 1.5 mm.

Subjects had two additional scans to generate quantitative MR data:

#### T2 mapping

Dual-echo CSF suppressed, FFT2 (fast FLAIR T2) data sets were acquired using TR/TI/TE1,2 = 5000/1638/15,120 ms. Twenty-eight contiguous axial slices were obtained with an acquisition matrix of 256 × 256 and a 24 cm in-plane field of view, giving a voxel size of 0.94 × 0.94 × 5 mm. T2 signal intensity was calculated pixel-by-pixel using the dual-echo data sets, one set from the early echo (TE_1_) and one from the late echo (TE_2_), according to the equation: T2 signal = (TE_2_ − TE_1_) / ln (*S*_1_/*S*_2_) where *S*_1_ and *S*_2_ represent the signal intensities for the early and late echoes respectively (Rugg-Gunn et al., 2005). Total acquisition time was 13 min and 50 s.

#### Magnetization transfer imaging

Magnetization transfer (MT)-weighted and non-MT-weighted three-dimensional data sets were acquired using TR/TE = 22.6/5.4 ms. Volume matrix was 256 × 256 (in-plane) × 124 over a 240 × 240 × 180 mm field of view, giving a voxel size of 0.94 × 0.94 × 1.5 mm. MT-weighted images were collected after the application of a pre-pulse to saturate the broad resonance of immobile macromolecule protons. The MT radiofrequency pulse used was a three-lobe Hamming apodized sinc pulse with a duration of 6.4 ms and of 5.1 μT, applied 2 kHz off-resonance (Rugg-Gunn et al., 2003). Total acquisition time was 13 min and 54 s.

### MRI quantitation

Voxel-by-voxel statistical comparisons were performed on each of the two image contrasts. This process used SPM99 (Wellcome Dept. of Imaging Neuroscience, Institute of Neurology, London, UK) to produce intensity probability maps that were normalized to a standard space defined by a template of all the controls (Salmenpera et al., 2007). The normalized T2 maps were smoothed with a 4 mm and MTR maps an 8 mm isotropic Gaussian kernel to improve signal to noise ratio, to allow the images to conform more closely to a Gaussian field model and to increase the validity of statistical inference. A standard univariate *t*-test was applied using SPM99 to every voxel in the image in order to create a map from which statistical inference was drawn. Significant increases or decreases in MR contrast signal intensity were detected at an individual voxel threshold of *p* < 0.001. The theory of Gaussian fields was used to calculate a corrected multiple comparison *p* value of 0.05. We tested for regional differences in intensity between each patient and the control group. Further details of the image processing are given in a previous paper (Salmenpera et al., 2007).

Quantitative MR values were obtained by overlaying a region of interest on the quantitative MR data. The mean intensity value of all voxels within the region of interest was calculated.

### MR:pathology visual correlation

All subjects had a standard anterior temporal lobe resection performed by the same surgeon. During and after resection, specimens were processed according to standard histopathological and co-registration protocols and digital images of the tissue blocks were correlated with the pre-operative volumetric MRI data (Eriksson et al., 2005). Briefly, after fixation the resected temporal lobe was sliced in 5 mm blocks perpendicular to the maximum linear extent of the superior temporal sulcus. The MR volume data were rotated and reformatted into an oblique coronal plane that matched the orientation of these blocks. These oblique coronal images were visually inspected in comparison to the pathological blocks. Using standardized criteria, the best fit MRI slice for any one pathological block was identified and matches for adjacent blocks of tissue ensued. Two raters (SF and SE) performed this task separately and reached a consensus in any case with discrepancy.

All subjects had post-operative MRI scans to indicate the extent of the resection.

### Histopathology

**Fig. 1:**
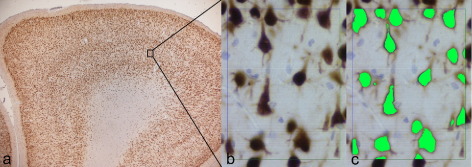
Example of images used for field fraction of NeuN immunohistochemistry. (a) Gyral crown of middle temporal gyrus at × 1.6 magnification (NeuN immunohistochemistry counterstained with H&E, 20 μm slice thickness). All cortical layers were included in the analysis. (b) Higher magnification (× 40) from the same specimen, used for field fraction analyses. Note that neuronal cell bodies, not only nuclei, are immunopositive, but there is only limited involvement of axons and dendrites. (c) Same field as in b with pixels immunopositive for NeuN highlighted in green. The RGB parameters representing immunopositive pixels (brown) are set in the first field analyzed.

Standard laboratory protocols were used to prepare resected tissue in sections stained for Nissl (cresyl violet/LFB), glial fibrillary acidic protein (GFAP; Dako, Cambridge, UK; polyclonal 1:1500) and neuronal nuclear antigen (NeuN; Chemicon, Temecula, CA, USA, monoclonal 1:2000). An experienced epilepsy neuropathologist (MT) made a qualitative tissue assessment for clinical purposes. For quantitative histopathology the following sections were used: the standard 7 μm GFAP sections; additional 20 μm sections stained with NeuN; 20 μm sections stained for microtubule associated protein (MAP2; SIGMA, Saint Louis, MO, USA; 1:2000); 7 μm sections stained with anti-synaptophysin antibody (Dako, Cambridge, UK; 1:100). All sections were processed in the same batch to ensure uniform immunostaining, which is important when assessing the field fraction, which relies on the intensity of staining. A commercial image analysis system (Histometrix, Kinetic Imaging, Liverpool, UK), attached to a Zeiss Axioskop microscope, was used to generate quantitative histopathology measures. Two regions of tissue were assessed in each subject: ROI1 in the deep white matter of the middle temporal gyrus (a region identified from MRI-directed correlation, see below); and ROI2, in the cortex in the gyral crown of the middle temporal gyrus. Quantitative measures in ROI1 were: stereological counts of neurons; field fraction estimates of immunostaining for GFAP, MAP2 and synaptophysin. Quantitative measures in ROI2 were stereological counts of neurons and field fraction estimates of staining for NeuN and GFAP. The stereological neuronal counts were performed as previously described (Eriksson et al., 2006). For field fraction estimates the ROI was outlined on the computer screen of the image analysis system at × 2.5 magnification. The number of fields within the ROI to be analyzed was chosen (70–90 in our study) and the computer software randomly chose a starting point and then a field spacing to cover the whole ROI at the relevant sampling rate. All cortical layers were included in the analysis. Magnification was then increased to × 40 and each field displayed on the computer screen. In the first field for analysis, light intensity parameters were optimized to visualize the immunopositive pixels and the RGB (red-green-blue) parameters representing immunopositive pixels were set. Light intensity and RGB parameters were kept constant for all subsequent fields. The software program then automatically estimated the number of immunopositive pixels as a proportion of the entire field. An average of field fraction for all fields was calculated, thus reporting a single field fraction value for each ROI in a given patient. RGB parameters were defined uniquely for each case before analyses as signal intensity can vary slightly between cases. An example of images used for field fraction analyses in one patient is shown in Fig. 1. Measures in ROI1 were made by an experienced epilepsy neuropathologist (MT) and measures in ROI2 by a neurologist (SE) with extensive experience of quantitative neuropathology.

### Histopathology and MRI correlation

#### MRI-directed

Abnormal regions in the SPM results for the MRI data were observed for several subjects (Table 1). To correlate MRI data with histopathological analyses in the same area in all cases, region of interest analyses were performed. A region that had abnormal T2 signal on voxel based analysis in one subject that was within the deep white matter of the middle temporal gyrus was selected and designated ROI1. This region was of sufficient size to provide robust histopathological data (Eriksson et al., 2006) and was present in tissue which had been resected in all 9 subjects. Since ROI1 and all quantitative MRI data used for the SPM analyses were normalized to SPM space, quantitative MTR and T2 data could be achieved by superimposing ROI1 on each case's normalized MTR and T2 data.

**Fig. 2:**
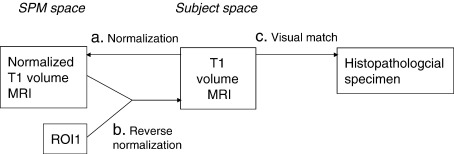
Normalization protocol. (a) Each subject's T1-weighted volume MRI scan was normalized to the T1 image template in SPM99, using the default normalization protocol. (b) The normalized T1-weighted image was hence in the same space as ROI1 and both could be reverse normalized to subject space using the parameters derived from the original normalization (a). (c) ROI1 could then be transferred to the equivalent histopathological section using the previously determined MRI:pathology correlation.

To achieve quantitative histopathological data, the ROI was aligned with the volume MRI of each patient by a sequence of two normalizations as follows (Fig. 2).

**Fig. 3:**
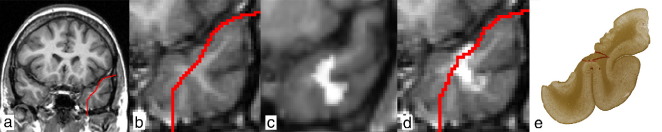
Example of placement of ROI1. (a) The original T1-weighted volume MRI in subject space with a line demarcating the resection. (b) Zoomed image of right temporal lobe on T1-weighted volume MRI seen in a. (c) Zoomed T1-weighted volume MRI after normalization into SPM space, with region of interest ROI1 shown overlaid in white onto the T1-weighted image. (d) Zoomed image of the T1-weighted volume MRI and ROI1 after the back normalization to subject space. The same normalization parameters were used for both the T1-weighted image and ROI1. (e) The equivalent histopathological slice which correlates with the MRI slice (GFAP, 7 μm slice-thickness), showing ROI1, outlined with red marker.

Each subject's volume MRI (cropped and tilted as used for the initial MR:pathology correlation) was normalized to the T1 image template in SPM99 using the default normalization protocol in SPM99 (Fig. 2). The T1 image template was in the same image space as ROI1, thus ROI1 could be associated with each subject's normalized MRI. The normalized volume was then back normalized to the volume MRI in each subject's native image space. The parameters used for the back-normalization were also used to transform ROI1 from T1 space back to the native space (Fig. 2). This ROI could then be transferred to the equivalent pathological space by visual inspection of the previously determined MRI:pathology correlation (Fig. 2). Thus ROI1 was located on the pathology slices of each subject. This region, directly drawn onto the pathology slides of interest, was used for subsequent quantitative histopathology. An example of the images of ROI1 for one subject is shown in Fig. 3.

We also defined a region in the pathological data and determined its MR characteristics. To achieve this, the MRI volume data were segmented in subject space using intensity thresholding and manual drawing in Analyze AVW 5.0 (BIR, Mayo Clinic, Minnesota). The entire resection volume and the middle temporal gyrus within the resection volume were extracted by manual drawing guided by visual inspection of the previously determined MR:pathology correlation. Intensity thresholding and visual inspection were used to separate gray and white matter. A final manual segmentation step was used to separate the gray matter of the gyral crown (ROI2), from the gray matter in the rest of the middle temporal gyrus. This step used similar criteria to characterize the limits of the gyral crown as were used by the neuropathologist in the quantitative neuropathology. The data from the initial MR:pathology correlation were used to determine which MR slices within the middle temporal gyrus in each subject corresponded to ROI2. Each subject's MR volume data were registered to the advanced MR image data in the native subject space using SPM99 and the default registration option. Because of the different qualities of the T2 maps and MTR image data, different registration was performed and different templates used that more closely resembled the two data sets.

**Fig. 4:**
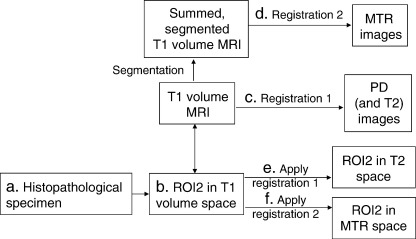
Registration protocol for ROI2. (a) The gyral crown of the middle temporal gyrus was identified on each patient's histopathological specimen (ROI2). (b) ROI2 was then determined on the corresponding T1-weighted volume scan using Analyze AVW 5.0. (c) The T1 volume scan was then registered to the PD images (and hence also the T2 images that were acquired simultaneously) using the SPM99 default registration options (registration 1). (d) The summed segmented T1 volume MRI (used to achieve image quality similar to MTR images) was registered to the MTR images using default options in SPM99 (registration 2). (e and f) The parameters from the two registrations were used to register ROI2 from T1 volume space to each patient's individual T2 and MTR space.

**Fig. 5:**
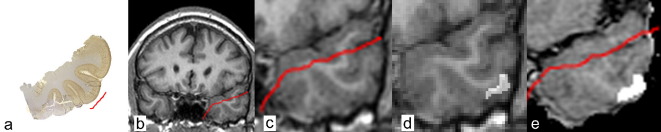
Example of placement of ROI2. (a) A histopathological slice used to estimate quantitative histopathology in the gyral crown of the middle temporal gyrus, indicated in red (NeuN, 20 μm slice thickness). (b) The corresponding volume MRI slice with a line demarcating the resection. (c) Zoomed image of the right temporal lobe on T1-weighted volume MRI seen in b. (d) The same image as in c with the region of interest ROI2 (i.e. the cortical crown of the middle temporal gyrus defined after segmentation of the MRI volume data set) overlaid. (e) Zoomed image of the right temporal lobe on MTR images overlaid by ROI2 after appropriate registration.

For the MTR images, the segmented gray and white matter (summed) were used, while the MR volume data were registered to the proton density image which was acquired simultaneously with the T2 data, thus effectively registering the MR volume to the T2 data. Once optimal registration parameters were identified, these were used to register ROI2 in the MR volume data to the T2 maps and MTR data sets (Fig. 4). Thus, for example, gray matter in the middle temporal gyrus could be identified on the T2 image and T2 values determined for the gray matter in that region. An example of the images of ROI2 for one subject is shown in Fig. 5.

Quantitative MR and histopathology measures were compared using SPSS (version 11.0.0).

## Results

Careful correlation of abnormal regions in the SPM data and the resected tissue indicated that regions that were abnormal on SPM analysis were physically present in the resected tissue in four patients (Table 1). In one patient (patient 3) qualitative neuropathology identified a hamartia (a subtle malformation of cortical development) in the white matter approximately 3 mm in diameter (Table 2). This was not detected using the SPM analysis and was not a part of ROI1 or ROI2. The abnormal region in one patient (T2 region in patient 5) was in the white matter of the middle temporal gyrus and was labeled as the region of interest, ROI1. The quantitative histopathology and quantitative MR data for this region for the nine patients are given in Table 3. Spearman Rho correlation did not reveal any significant correlations between any of these pathological and MR measures. This region had an abnormal average T2 value in one other patient (patient 1) but MT values were within the normal range for all subjects. Clustering analysis of the histopathological data could not separate the two patients with abnormal T2 values from those subjects with normal T2 values in ROI1.

Quantitative histopathology was determined for the gray matter in the gyral crown of the middle temporal gyrus. The registration process enabled us to determine quantitative MR data for the same region (ROI2) (Table 4). There was a significant Spearman Rho negative correlation between the field fraction measure of NeuN and the T2 value in ROI2 (correlation coefficient − 0.72, *p* = 0.028).

## Discussion

We have previously shown that in vivo MRI and pathological blocks may be aligned using visual inspection and a standardized protocol (Eriksson et al., 2005). We have now shown that, with additional registration protocols, quantitative parameters from MRI and histopathology can be directly compared. There was a negative correlation between the histopathological field fraction measure of NeuN immunopositivity and the MR measure of T2 relaxation time in the gray matter of the middle temporal gyrus. This is the first correlation between a quantitative MR parameter and histopathological data from neocortex in patients with epilepsy.

We wished to compare the same area in all patients because it is not known how much histopathological measures and MR parameters differ as a function of location within cerebral tissue. Although some of the histopathological features of interest were readily quantified with a semi-automated protocol, stereological measures require significant expert time input. In consequence, we restricted this study to two regions of interest. The first region investigated corresponded to a region of abnormal T2 signal on SPM analysis in one patient and was chosen since it was of sufficient size to provide robust histopathological data and was available in all resections. Our second region was identified in each subject's histopathological sections, the gray matter of the crown of the middle temporal gyrus overlying ROI1, giving us data in adjacent gray matter.

The limitation of the initial MR and pathology matching is that the matching was visual and was guided by anatomy and was to some extent subjective. Our previous study, however, showed that the protocol gave consistent, reliable and accurate registrations within the structural limitations of the histopathological data (Eriksson et al., 2005). The subsequent matching of specific MR or pathology regions with their respective pathology or MR regions was carried out using computational normalization and registration, both of which were entirely repeatable. This may seem a complex procedure when, for example, the middle temporal gyrus is generally readily identified on anatomical MRI. However, the advanced quantitative MR sequences are optimized for the appropriate MR parameter and not for visualization of anatomy and it can therefore be difficult to confidently identify anatomical features using these contrasts. Identifying the structure of interest in a high quality anatomical scan and then using registration and/or normalization protocols reduced the number of subjective steps (only one definition of the tissue structure was required rather than one in each MR scan) and therefore reduced computation time and increased repeatability.

The T2 signal in MRI is dependent upon the relationship between bound and free water in a tissue, which is itself dependent upon the macromolecular environment. Disruption of tissue integrity can result in an increase in the free water in tissue and an increase in T2 relaxation time. Changes in T2 in the cerebrum have been related to, among other processes, gliosis, demyelination, edema, neuronal loss and infarction. In an earlier study from our group, significant increases in T2 signal were observed in 51% of patients with cryptogenic focal epilepsy (Rugg-Gunn et al., 2005). The MTR signal is dependent upon the exchange of magnetization between free and bound protons in tissue and this depends upon the integrity of the macromolecular environment. Changes in MTR have been related to histopathologic changes of axonal loss, gliosis, demyelination and edema (Lexa et al., 1994; Brochet and Dousset, 1999). Our earlier study found reductions of MTR in acquired lesions, malformations of cortical development and in regions that were normal on conventional MRI (Rugg-Gunn et al., 2003). In the present study ROI1 was defined by an abnormal T2 value in one subject. Additional analysis revealed that this region also had an abnormal T2 signal in one other subject. MTR values were normal in this region in all subjects. There were no significant linear correlations between the MR values and the histopathological values in ROI1 in our group. The two subjects with abnormal T2 values did not differ from the other seven subjects in relation to their standard or quantitative histopathological data. The T2 and MTR values were all normal in ROI2.

There was a significant negative correlation between the T2 relaxation time and the field fraction estimate of NeuN staining in the cortex of the middle temporal gyrus. A decrease in NeuN staining could suggest a reduction in the number of mature neurons in the tissue, altered packing density or alterations in cell size or NeuN immunoreactivity. An increase in T2 signal has been correlated with neuronal loss and increased gliosis (Van Paesschen et al., 1997; Rugg-Gunn et al., 2005). Increased gliosis may further reduce the relative concentration of neurons in a region, although sometimes, tissue sclerosis or gliosis causes reduction in tissue volume which could paradoxically increase packing density. We did not observe any relationship between T2 value and GFAP field fraction. Thus, the correlation detected may reflect a consistent biological relationship focused on neuronal loss. However, one subject had neocortical neuronal loss observed during routine qualitative neuropathology (Case 4). This subject had the lowest value of neuronal count in the gray matter of ROI2 (the crown of the middle temporal gyrus) but also the lowest value of T2 in ROI2 which appears contrary to the correlation observed in the whole group. The reason for this is not clear. The cortical NeuN field fraction in this case was 0.11 (range 0.08–0.13).

Gray matter neuronal counts did not correlate with the field fraction estimate of NeuN. The gray matter neuronal counts are a count for each neuron observed in the region of interest expressed as a proportion of the total area assessed. The field fraction estimate of NeuN is an estimate of the area occupied by tissue immunopositive for NeuN as a proportion of the total area assessed. This tissue is predominantly neuronal cell bodies with some axons and dendrites. Thus 5 large neurons will occupy a greater area of immunopositivity than 5 small neurons. The neuronal count would be the same in each case but the field fraction would be different. This may explain the lack of correlation between the field fraction and the neuronal count. The relation of these two measures with the T2 relaxation value suggests that the T2 relaxation is dependent on more than just the number of neurons, also on their size or complexity, which may reflect their function. Further staining protocols and analyses of neuronal size are necessary to elucidate further this difference.

The lack of any other correlations between the MR and the histopathology measures could be due to a number of factors. A real correlation may exist and not have been detected, possibly because of the limited number of subjects in the study. A change of MR scanner curtailed data collection for this series and we are now recruiting more subjects imaged using a new 3 T scanner. We could also have missed a true correlation because the registration between the MR region and the pathological region was inadequate as discussed previously.

Alternatively, there may not be any other true correlations between the MRI sequences and pathological measures studied. The pathological measures characterize real physical features — for example, the actual number of neurons and the proportion of tissue affected by gliosis. However, the underlying physical significance of the MR data is not yet well characterized. We may have chosen pathological measures which do not relate to the features characterized by the MR signals. Myelin content, for example, might influence both T2 and MTR. We did not think it likely that myelin studies would be of relevance in epilepsy in the context of this paper, but additional histopathological analyses, such as LFB for myelin or myelin basic protein, may lead to further useful data in future studies.

There was also limited tissue specificity for absolute T2 and MTR values between white and gray matter (see Tables 3 and 4 for details). Thus the MR measures and the histopathology measures may be measuring different features of the tissue and would not be expected to correlate. A hamartia, seen as a cluster of neurons in the white matter microscopically, was not detected as an abnormal MR region with either MTR or T2 using SPM. There are new histopathological techniques in development and some of these may prove a better match to the MR signal changes. MR acquisition on a 3 T scanner will produce data with better signal-to-noise qualities and improved spatial resolution and may increase the sensitivity of quantitative MR measures. Further correlations of quantitative histopathology and quantitative MRI measures may lead to derivation of MRI measures that are most sensitive to pathological changes.

Our protocol for matching MR and pathology has demonstrated how we can investigate both the histopathology directed by an MR finding and the MR data directed by a histopathological finding. We identified a biologically plausible correlation between an increased T2 relaxation time and a reduction in neuronal labeling in the cortex of the middle temporal gyrus. If newer, quantitative MRI sequences are to be adopted more widely in routine clinical practice, further research exploring their underlying biological and pathological substrates will be necessary and may ultimately lead to a reduction in the proportion of patients with drug-resistant focal epilepsy whose optimal MRI is said to be “normal”.

## Figures and Tables

**Table 1:** Surgical and quantitative MRI data (within the resected tissue)

Subject	Gender	Age	TLx	MTR	T2	Outcome
1	M	36	R	Dec	Inc	1
2	M	35	R			2
3	F	44	R			1
4	M	28	R			3
5	F	46	R		3 Inc	1
6	F	31	R			4
7	F	40	R		Inc	1
8	F	37	L		2 Inc	3
9	M	33	L			1

TLx: side of temporal lobe surgery; R: right; L: left; Inc: increased signal compared to controls identified using SPM; MTR: magnetization transfer ratio; Dec: decreased signal compared to controls; 2 Inc: 2 regions of increased signal etc. Outcome according to ILAE criteria (Wieser et al., 2001) after a minimum of 1 year: 1 no seizures; 2 auras only; 3 seizures on less than 4 days a year; 4 seizures reduced by more than 50%.

**Table 2:** Qualitative histopathological data in the complete resection specimen (whole temporal lobe and hippocampal specimens)

Subject	Diagnosis
1	DNT
2	Hamartia in white matter, approximately 2 mm diameter, insufficient hippocampal material to allow confirmation of classic HS, but no evidence of neuronal loss or gliosis in CA1
3	Hippocampal gliosis—no classical HS, marked cortical gliosis and moderate white matter atrophy
4	HS
Possible neuronal loss from outer cortical layers with abnormal neuronal orientation, moderate superficial cortical gliosis and white matter atrophy
5	HS
Prominent superficial cortical and white matter gliosis
6	Hippocampal gliosis, insufficient hippocampal material to allow confirmation of classic HS, gliosis of entorhinal cortex and neocortex
7	HS, moderate superficial cortical and white matter gliosis
8	Ganglioglioma, patchy moderately severe gliosis in deeper cortical layers
9	HS, neuronal loss and excessive gliosis in superficial cortical laminae in anterior temporal lobe

DNT: dsyembryoplastic neuroepithelial tumor; HS: hippocampal sclerosis.

**Table 3:** Quantitative histopathology and quantitative MR for white matter (ROI1), in all subjects

Subject	MAP2	Synaptophysin	GFAP	WMNeu	T2	MTR
1	0.17	0.09	0.53	4.17	111	39
2	0.14	0.14	0.22	4.06	104	43
3	0.26	0.09	0.30	2.40	99	43
4	0.19	0.06	0.35	2.18	99	43
5	0.21	0.12	0.23	2.76	119	49
6	0.15	0.06	0.32	0.98	102	43
7	0.18	0.06	0.45	2.28	102	45
8	0.17	0.12	0.39	2.53	107	44
9	0.20	0.03	0.69	1.37	109	46

Values of MAP2, Synaptophysin and GFAP are all field fraction proportions for MAP2, synaptophysin and GFAP respectively. WMNeu: white matter neuronal density, neurons × 10^− 6^/μm^3^. T2 relaxation time (ms). MTR p.u. (percentage units). MAP2: microtubule associated protein 2; GFAP; glial fibrillary acidic protein; MTR: magnetization transfer ratio; SD: standard deviation. Mean difference between repeat measurements for field fraction for MAP2, 0.037 (SD = 0.042); synaptophysin, 0.011 (SD 0.2); GFAP, 0.1 (SD = 0.13). Mean T2 value in this ROI for control subjects was 99 (SD 3.1, coefficient of variation 3.1%) and mean MTR for control subjects was 49 (SD 2.2, coefficient of variation 4.5%).

**Table 4:** Quantitative histopathology and quantitative MR for gray matter (ROI2), in all subjects

Subject	GFAP	GMNeu	NeuN	T2	MTR
1	0.09	4.31	0.08	117	40
2	0.04	4.20	0.12	111	44
3	0.11	6.16	0.13	102	46
4	0.17	2.89	0.11	92	47
5	0.15	3.89	0.08	118	46
6	0.05	3.86	0.11	110	47
7	0.12	4.04	0.10	105	46
8	0.09	4.05	0.10	118	40
9	0.10	3.17	0.10	112	45

Values of GFAP and NeuN are all field fraction proportions as in Table 3. GMNeu: gray matter neuronal density, neurons × 10^− 5^/μm^3^. T2 relaxation time (ms). MTR p.u. (percentage units). GFAP: glial fibrillary acidic protein; NeuN: neuronal nuclear antigen; MTR: magnetization transfer ratio. Mean of difference for repeat measures of field fraction for GFAP, 0.06 (SD 0.06); NeuN, 0.012 (SD 0.007). Mean T2 value in this ROI for control subjects was 101 (SD 3.5, coefficient of variation 3.5%) and mean MTR for control subjects was 46 (SD 1.8, coefficient of variation 3.9%).
